# Advancements in the Treatment of CLL: The Rise of Zanubrutinib as a Preferred Therapeutic Option

**DOI:** 10.3390/cancers15143737

**Published:** 2023-07-23

**Authors:** Stefano Molica, Constantine Tam, David Allsup, Aaron Polliack

**Affiliations:** 1 Queens Centre for Oncology and Haematology, Castle Hill Hospital, Hull University NHS Trust, Hull HU16 5JQ, UK; david.allsup@hyms.ac.uk; 2 Alfred Hospital, Melbourne, VIC 3004, Australia; 3 Centre of Biomedicine, Hull York Medical School, University of Hull, Hull HU16 5JQ, UK; 4 Department of Hematology, Hadassah-Hebrew University Medical Center, Jerusalem 91120, Israel; aaron.polliack@mail.huji.ac.il

**Keywords:** CLL, zanubrutinib, BTK inhibitors, efficacy, safety, chronic lymphocytic leukemia

## Abstract

**Simple Summary:**

Due to improved selectivity and favorable toxicity profiles, the next-generation Bruton’s tyrosine kinase inhibitors (BTKis) are replacing ibrutinib in the treatment of B-cell malignancies including chronic lymphocytic leukemia (CLL). While efficacy between different BTKi agents is probably similar, there are important differences in toxicity profiles (including lower incidences of cardiovascular complications) that favor the choice of second-generation BTKis such as zanubrutinib. Updates in the National Comprehensive Cancer Network (NCCN) guidelines and German CLL treatment algorithm and approvals from both the Food and Drug Administration (FDA) and European Medicines Agency (EMA) support zanubrutinib as a preferred option for the treatment of both treatment-naïve and relapsed/refractory CLL patients irrespective of patient fitness.

**Abstract:**

Ibrutinib, the first-in-class Bruton’s tyrosine kinase inhibitor (BTKi), is a commonly deployed therapeutic option for previously untreated and relapsed/refractory (R/R) patients with chronic lymphocytic leukemia (CLL). The use of ibrutinib is, however, partially limited by off-target side effects. Zanubrutinib (zanu) is a second-generation BTKi with enhanced target selectivity and occupancy of the kinase binding site. The SEQUOIA study showed that zanu significantly prolonged progression-free survival (PFS) when compared to bendamustine–rituximab (BR) in treatment-naive CLL patients. More recently, data from the phase III ALPINE trial, which directly compared zanu with ibrutinib, demonstrated that zanu’s advantages include an improved safety profile as well as enhanced clinical efficacy. Based on the results of the SEQUOIA and ALPINE pivotal trials, the Food and Drug Administration (FDA) and European Medicines Agency (EMA) licensed zanu for the treatment of patients with CLL or small lymphocytic lymphoma (SLL) in January 2023. The updated (v2.2023) National Comprehensive Cancer Network (NCCN) guidelines and the most recent German CLL algorithm suggest that zanu may replace first-generation BTKis as a preferred therapeutic option for patients with CLL/SLL due to its increased selectivity for the kinase binding site, improved therapeutic efficacy, and favorable toxicity profile. Some drug class-related characteristics such as drug resistance, low complete remission (CR) rates, and indefinite treatment duration still remain with zanu, and the results from recently completed and ongoing fixed-duration clinical trials, combining zanu with an anti-BCL2 agent, are eagerly awaited with the possible promise of a reduced treatment duration and lower financial burden.

## 1. Introduction and Background

The inhibition of Bruton’s tyrosine kinase (BTK) has provided an array of therapeutic options for the effective treatment of chronic lymphocytic leukemia (CLL) [1,2]. In 2014 the first-in-class oral BTK inhibitor (BTKi) ibrutinib was licensed and represented a major advance in CLL treatment. Responses to treatment with ibrutinib were favorable in patients with high-risk CLL who had relatively poor response rates to chemo-immunotherapy (CIT) in the past [3,4,5,6,7,8,9]. BTKi treatment, though highly effective, does not generally achieve deep responses in terms of low levels of measurable residual disease (MRD), which results in a therapeutic paradigm where indefinite, continuous treatment is required to maintain clinical responses. Consequentially, patients are exposed to BTKi therapy for prolonged periods and are therefore prone to developing therapy-related toxicities, which include bleeding, infections, diarrhea, arthralgias, arrhythmias, and hypertension [10].

Randomized clinical trials have investigated the efficacy of second-generation BTKis such as acalabrutinib and found reduced toxicity when compared with ibrutinib. These improvements in the safety profile are attributed to the greater selectivity of the second-generation BTKi’s, which as a class have reduced off-target kinase inhibition compared with ibrutinib. In a phase III open-label, randomized, prospective study (ELEVATE-RR trial), acalabrutinib had equivalent efficacy but enhanced safety compared to ibrutinib, with fewer episodes of atrial fibrillation and lower rates of drug discontinuation due to side effects [11]. Accordingly, acalabrutinib was the first covalent, second-generation BTKi to receive Food and Drug Administration (FDA) and European Medicines Agency (EMA) approval for CLL treatment [12,13].

Zanubrutinib (zanu) is another next-generation small molecule BTKi that forms a covalent bond with cysteine residues in the BTK active binding site, leading to potent inhibition of kinase activity [14]. Zanu was initially approved for the treatment of patients with relapsed mantle cell lymphoma (MCL) and relapsed/refractory (R/R) marginal zone lymphoma (MZL) based on the results of single-arm studies. Subsequently, it received approval for the treatment of Waldenstrom’s macroglobulinemia (WM) based upon the results of the phase 3 ASPEN study, which compared the efficacy and safety of zanu with ibrutinib, and showed reduced toxicities in favor of zanu [15,16,17,18]. Finally, in January 2023, the FDA and EMA approved zanu for the treatment of patients with CLL or small lymphocytic lymphoma (SLL) based on the results of the pivotal phase 3 SEQUOIA (NCT03336333) and ALPINE (NCT03734016) trials [19,20,21,22]. Due to the increased selectivity, improved efficacy, and superior toxicity profile of zanu, this agent is now listed as the preferred treatment option for patients with CLL in the updated National Comprehensive Cancer Network (NCCN) (v2.2023) guidelines and recently released German algorithm [23,24].

## 2. Earlier Studies of Zanubrutinib in CLL

The first-in-human, open-label, multicenter, phase I/II study of zanu (AU-003 study) included R/R patients with B-cell malignancies who received the drug at doses of 40, 80, 160, or 320 mg once daily or 160 mg twice daily, and safety, tolerability, and maximum tolerated dose were the primary endpoints [25]. The expansion study enrolled a further 94 treatment-naïve (TN) or R/R CLL/SLL patients who received zanu at the maximum tolerated dose until disease progression, loss of clinical benefit, or dose-limiting toxicity. Patients were given zanu at doses of 160 mg twice daily (81 patients), 320 mg once daily (40 patients), or 160 mg once daily (40 patients). After a median follow-up of 13.7 months, 89 patients with CLL/SLL (94.7%) were still on the study. The overall response rate (ORR) was 96.2% for 78 evaluable patients, while the estimated 12-month PFS was 100%. Most of the toxicities were grade 1/2; neutropenia was the only grade 3–4 toxicity observed in more than two patients, while a grade 3 subcutaneous hemorrhage occurred in only one patient [25]. These relatively favorable safety results were in keeping with the reduced affinity of zanu for off-target kinases (including epidermal growth factor receptor (EGFR), Janus kinase 3 (JAK3), human EGFR2, interleukin-2 (IL2), inducible T-cell kinase (ITK), and TEC) compared to ibrutinib [26].

Cull et al. [27] recently reported updated safety and efficacy data of the AU-003 study involving 123 patients with a median follow-up of 47.2 months. The ORR was 95.9% (TN, 100%; R/R, 95%), with 18.7% achieving a complete response (CR). In 16 patients with del(17p)/*TP53* mutation, the ORR was 87.5% (CR 16.7%). The estimated 3-year PFS was 83% (TN, 81%; R/R, 83%, respectively). Discontinuation of the drug due to adverse events (AEs) or disease progression were rare. The efficacy results of this updated analysis are also consistent with those of a single-arm Chinese study (NCT03206918) that reported an ORR of 84.6% (CR 3.3%) in 91 R/R CLL patients, with 87.2% of patients still alive and progression-free at 12.9 months. In relation to long-term safety, the results of the AU-003 study indicate that neutropenia (15.4%), pneumonia (9.8%), hypertension (8.9%), and anemia (6.5%) were the most commonly reported Grade 3 AE, while the annual incidence of atrial fibrillation, major hemorrhage, grade 3 neutropenia, and grade 3 infection decreased over time [27]. The latter study, which included a significant proportion of patients with TN and R/R CLL/SLL treated with single-agent zanu for 4 years, provided significant evidence of its long-lasting efficacy and safety.

Overall, data generated in these phase I/II studies demonstrated that:

1. The twice-daily dosing of zanu achieves 8-fold higher plasma drug exposure than ibrutinib and a longer half-life than acalabrutinib (4 vs. 1 h) [25,28].
2. Zanu shows complete and sustained occupancy of the BTK binding site across lymph nodes and in peripheral blood mononuclear cells [25,28].
3. Consistent with the favorable oral bioavailability evident in preclinical studies, oral administration of zanu achieves therapeutic plasma drug concentrations using the recommended phase II dose of 160 mg twice daily, with maintenance of drug levels above the IC_50_ required for full occupancy of the BTK binding site [25,28].
4. Zanu is less prone to pharmacological interactions with food, drug–drug interactions with strong or moderate CYP3A inhibitors, and proton pump inhibitors (PPIs) leading to more consistent, sustained therapeutic exposures and improved dosing convenience. In addition, the clinical use of zanu is less sensitive to impairments of liver function than ibrutinib [29].

## 3. Phase III Clinical Trials of Zanubrutinib in CLL

The recent FDA and EMA approvals of zanu for the treatment of CLL/SLL patients were based on the results derived from the phase III SEQUOIA and ALPINE trials, which demonstrated significant therapeutic efficacy and a favorable safety profile for zanu in both the first-line and the R/R setting.

### 3.1. Sequoia Trial

**Figure 1:**
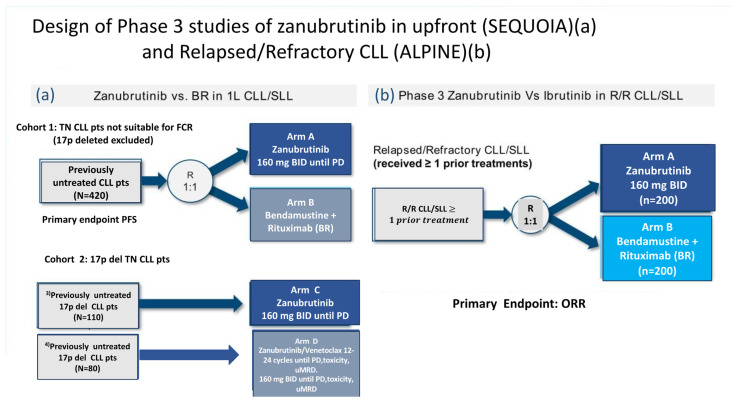
Design of phase III studies of zanubrutinib in treatment-naive (SEQUOIA) (**a**) and relapsed/refractory CLL (ALPINE) (**b**) ([19,20], ^3^ [31], ^4^ [32]).

SEQUOIA was a randomized, multicenter, global, phase III trial (NCT03336333) assessing the efficacy and safety of zanu in patients with TN CLL or SLL. This trial consisted of three cohorts (Figure 1a) [30]:

- Cohort 1 comprised 479 patients without del(17p) who were randomized 1:1 and either assigned to receive zanu (*n* = 241)(until disease progression or unacceptable toxicity) or to bendamustine and rituximab (BR) (*n* = 238) for up to six cycles.
- Cohort 2 comprised 110 patients with del(17p) who were assigned to receive zanu monotherapy as it was deemed unethical to randomize patients with del(17p) to BR.
- Cohort 3 comprised 80 patients with del(17p) or *TP53* aberrations who were assigned to receive zanu in combination with venetoclax (ZV). This cohort was opened when Cohort 2 was fully enrolled in order to provide non-randomized treatment for patients with del(17p).

Cohort 1 of the SEQUOIA trial enrolled CLL patients who were ineligible for fludarabine, cyclophosphamide, and rituximab (FCR) due to age (65 years or older) or associated comorbidities (CIRS > 6, creatinine clearance <70 mL/min or history of serious infections). The primary endpoint, as assessed by an independent review committee, was PFS in the intention-to-treat population [19]. With a median follow-up of 26.2 months, the estimated 24-month PFS rates for the zanu and BR groups in Cohort 1 were 85.5% and 69.5%, respectively. In prespecified subgroup analyses, PFS was consistently superior with zanu compared to BR, regardless of age, gender, or high-risk disease status (such as Binet stage C, bulky disease, or *IGHV* unmutated status).

Indirect treatment comparisons between first- and second-generation BTKis may be limited by cross-trial differences; however, the outcomes of patients assigned to receive zanu in the SEQUOIA study, ibrutinib as a single agent in the ALLIANCE trial, and acalabrutinib in the ELEVATE-TN trial were all the same (PFS 87% at 24 months) [5,19,33]. With respect to the side effects encountered with BTKis, it is noteworthy that atrial fibrillation of any grade occurred in only 3% of patients treated with zanu, which is significantly lower than the 12.6% rate reported with ibrutinib in the ALLIANCE trial. On the other hand, the 5% major bleeding rate observed with zanu was equivalent to that recorded for other BTKis [5,19,33]. The rate of discontinuation of zanu due to adverse events (AEs) was also relatively low, at 13.7% [33]. Finally, the measurement of patient-reported outcomes (PROs) within the SEQUOIA study indicated that zanu is associated with a greater improvement IN Health-Related Quality of Life (HRQoL) compared to BR [34].

The well-known benefit associated with BTKi treatments over CIT-based therapies could minimize the value of the results obtained with zanu in comparison with BR in the context of the SEQUOIA trial [3,4,5,6,33]. However, SEQUOIA trial enrollment began in 2017 before the widespread availability of data showing that BTKi-based therapies outperformed CIT in TN CLL. Hopefully, the results of the efficacy and safety of zanu will be validated in future studies enrolling patient populations not included in the SEQUOIA trial such as those with a higher comorbidity burden or fit for FCR. Moreover, well-designed head-to-head trials comparing zanu with other second-generation covalent or non-covalent BTK inhibitors, such as pirtobrutinib, are needed to optimize the choice of optimal frontline BTKi therapy in the future.

In the non-randomized arm C of the SEQUOIA trial, which enrolled TN, del(17p) CLL/SLL patients, the median PFS and OS were not reached, with an 18-month PFS and OS of 88.6% and 95.5% respectively. Four patients in this cohort (3.7%) discontinued treatment due to AEs, while atrial fibrillation/flutter was reported in only 2.8% of patients [31]. Thus, the results obtained in arm 3 of the SEQUOIA trial in patients with del(17p) compare favorably with those of studies enrolling standard-risk patient cohorts [34].

The SEQUOIA trial also included an arm, Cohort D, in which patients with del(17p) received ZV with the discontinuation of treatment when a deep response was achieved based upon the attainment of CR and undetectable MRD (uMRD). This arm was designed and initiated when the arm C cohort completed enrollment. The ORR at a median follow-up of 12.0 months was 97.2% in 36 evaluable patients. Preliminary safety data also indicate that ZV was well tolerated, with no cases of clinical tumor lysis syndrome (TLS) and relatively low incidences of neutropenia (all grades, 20.6 %), diarrhea (14.7%), and nausea (14.7%). In addition, 38.2% of ZV patients experienced at least one AE at grade 3 or greater. AEs were responsible for dose interruptions in 29.4% of ZV patients, but there was no need for dose reduction or treatment discontinuation and there were no fatal AEs [32]. Longer follow-up is still needed, however, to fully assess the depth of response and the safety of ZV in this high-risk population of CLL patients.

### 3.2. Alpine Trial

The ALPINE trial was a randomized phase III study comparing the second-generation BTKi zanu to the first-generation BTKi ibrutinib (Figure 1b). This study was designed on the assumption that complete/sustained occupancy of the BTK binding site by zanu may improve efficacy outcomes and minimize off-target inhibition-related toxicities due to its increased specificity for BTK. In both arms, the study treatment was given to 652 patients with R/R CLL until disease progression or intolerance. In the ALPINE study, enrolled patients received a median of one prior line of therapy, and approximately 23% of participants had a 17p deletion or *TP53* mutation [35].

The primary endpoint of the ALPINE study was ORR-defined as either CR or partial response (PR). The first interim report of ALPINE showed that zanu was associated with a significantly improved ORR compared to ibrutinib (78.3% vs. 62.5%) [35]. The superiority of zanu over ibrutinib in terms of ORR was also confirmed when PR with lymphocytosis was included in the definition of ORR (89.9% vs. 82.5%). These results paved the way for the assessment of PFS differences in the ALPINE trial by a hierarchical statistical analysis [20,35].

In the PFS analysis, zanu significantly prolonged PFS compared to ibrutinib, as assessed by both the independent review committee and the investigators (hazard ratio (HR) for disease progression or death, 0.65). Furthermore, even in the highest-risk del(17p) and/or aberrant *TP53* patient group, a preplanned analysis showed that zanu improved the PFS by 22% [20,36]. One potential explanation for the superior efficacy of zanu observed, may be related to its more favorable pharmacokinetic properties, with persistence of zanu above the IC50 of BTK throughout the entire dosing interval, thereby providing continuous coverage against newly synthesized BTK in the CLL cells [37].

In terms of toxicity and the duration of treatment, fewer patients on zanu discontinued treatment due to AEs, and fewer discontinued zanu due to progressive disease compared to the ibrutinib-treated group. With a median follow-up of 29.6 months of treatment, the rate of treatment cessation was lower for zanu (26.3%) than ibrutinib (41.2%), with most discontinuations due to AEs (16.2% vs. 22.8%) or progressive disease (7.3% vs. 12.9%). These rates were somewhat higher than expected compared to those reported in previously published studies [34]. This may relate to the fact that the ALPINE study was an international clinical trial, enrolling patients from many countries, and may better reflect real-world situations in terms of BTKi being discontinued for AEs [20].

In the ALPINE study zanu patients had fewer serious AEs and serious cardiac AEs leading to drug discontinuation compared to ibrutinib-treated patients (1 vs. 14). Notably, no cardiac deaths were observed in zanu-treated persons vs. six in the ibrutinib group. Atrial fibrillation or flutter of any grade was reduced in the zanu-treated group compared to those in the ibrutinib group (5.2% vs. 13.3%) with reduced atrial fibrillation or flutter at grade 3 or higher (2.5% vs. 4.0%). Similar rates of hypertension were reported in the zanu (23.5%) and ibrutinib groups (22.8%), while neutropenia of any grade was recorded in 29.3% of patients in the zanu group vs. 24.4% of those in the ibrutinib group. Infections of any grade were documented in 71.3% of patients receiving zanu compared to 73.1% in the ibrutinib group, while rates of grade 3 or higher of infections were 26.5% and 28.1%, respectively [20].

In terms of how representative the results of the ibrutinib arm in ALPINE were, we note that the 18-month PFS of patients treated with ibrutinib in the RESONATE trial and of those who received ibrutinib in the ALPINE trial was similar (78% vs. 75%) [3,20]. However, different patient populations and different stratification factors make cross-trial comparisons difficult for, e.g., patients enrolled in the RESONATE trial had received more lines of prior therapies and included more cases of high-risk CLL (17p deletion) than individuals enrolled in the ALPINE trial [3,20]. Finally, in the ALPINE trial, patients with R/R CLL/SLL treated with zanu monotherapy reported more improvements in key HRQOL endpoints than those receiving ibrutinib monotherapy [38].

## 4. Specific Aspects of Zanubrutinib Therapy in CLL

### 4.1. Is It Possible to Simplify the Zanubrutinib Treatment Schedule?

Zanu dose selection has been a matter of contention during the clinical development of this drug. This is an important issue as the simplification of the zanu dosing regimen to 320 mg once daily instead of 160 mg twice daily may well improve drug adherence and thereby maintain overall dose intensity. To better understand the outcomes of different dose regimens of zanu in MCL, Ou et al. reviewed data from a single-arm, open-label multicenter phase II study in which patients were treated with zanu at 160 mg twice daily until disease progression or unacceptable toxicity [39]. The investigators also assessed data from the AU-003 first-in-human study of zanu administered in dose increments of 40 mg, 80 mg, 160 mg, or 320 mg once daily or 160 mg twice daily in patients with B-cell malignancies. A total of 86 patients were enrolled in the phase II study and 37 in the phase I study (of whom 32 were treated at the recommended phase 2 dose of either 320 mg once daily (*n* = 18) or 160 mg twice daily (*n*= 14)). For both dosing regimens, the median BTK occupancy in peripheral blood mononuclear cells was 100% across all time points. However, the median BTK occupancy in nodal tissue was higher for 160 mg twice daily than 320 mg once daily (100% vs. 93%); there were no notable differences in the safety and tolerability profiles of the two zanu dosing schedules. Overall, a similar degree of plasma exposure and BTK inhibition was achieved with the two zanu doses; thus, any differences in the trough and maximum plasma concentrations between the two regimens are unlikely to have a meaningful impact on efficacy and safety endpoints [39]. In contrast, Shadman et al. suggested that there may be a difference in the efficacy and toxicity of different zanu schedules used in patients with BTKi intolerance [40]. However, the relatively small number of patients included in these studies and the short periods of follow-up prevent firm conclusions being drawn in relation to the relative efficacy and safety of the 320 mg once daily zanu regimen. Thus, the issue of whether the once daily dose has an impact on efficacy or adverse effects needs to be carefully addressed in future post-marketing or real-world studies.

### 4.2. Zanubrutinib after Discontinuation of a Covalent BTKi Because of Toxicity

A real-world analysis of ibrutinib treatment in CLL revealed that 21% of treated patients discontinued this drug due to toxicity [41]. Although acalabrutinib has a greater selectivity for BTK than ibrutinib, this agent and its metabolite M27 continue to bind to other kinases during therapy leading to adverse events with the potential for the subsequent discontinuation of treatment. In a phase I/II study of acalabrutinib in CLL patients, 9% of participants discontinued treatment due to adverse effects [42]. Although acalabrutinib is a safe and effective option for patients with R/R CLL who cannot tolerate ibrutinib, patients who are intolerant of either ibrutinib or acalabrutinib currently have very few BTKis treatment options [43].

Shadman and colleagues [40] recently published the results of a phase II study of zanu treatment in 67 patients with B-cell malignancies (CLL/SLL, MCL, or MZL) who were previously intolerant to a BTKi. Fifty-seven of these patients were intolerant to ibrutinib, while 10 were intolerant to acalabrutinib. After a median duration of 11.6 months of zanu treatment, most of the prior intolerance events (81 of 115 (70%) for ibrutinib; 15 of 18 (83%) for acalabrutinib) did not recur with the zanu therapy. Of the recurrent intolerance events, seven (21%) of 34 ibrutinib and two (67%) of three acalabrutinib intolerance events recurred with the same degree of severity with zanu, while 27 (79%) ibrutinib intolerance events and one (33%) acalabrutinib intolerance event recurred at a lower severity with zanu. Among 64 efficacy-evaluable patients, the disease control rate was 93.8% and the ORR was 64.1% with an 18-month PFS of 83.8% [40]. This study is the first clinical trial to assess the safety and efficacy of the next-generation BTK inhibitor, zanu, in patients with previously treated B-cell malignancies intolerant to ibrutinib, acalabrutinib, or both. These results suggest that patients who are unable to tolerate ibrutinib and/or acalabrutinib or both may benefit from switching to zanu [40].

### 4.3. Combining Zanubrutinib with Monoclonal Antibodies or Anti-BCL2 Agents

Due to its substantial toxicity and other theoretical concerns, ibrutinib may not be the ideal BTKi to use in combination with an anti-CD20 antibody for the treatment of CLL/SLL [5,44]. In this respect, zanu may be a better option to use in combination, as it does not inhibit interleukin-2 inducible T-cell kinase (ITK), which is essential for the antibody-dependent cell cytotoxicity (ADCC) induced by anti-CD20 antibodies. In a phase I study by Tam et al., zanu was used in combination with obinutuzumab (ZO) to treat patients with CLL/SLL [45]. The ORR to ZO was 100% (*n* = 20) in patients with TN and 92% (*n* = 23) in patients with R/R CLL/SLL. Upper respiratory tract infection (51%) and neutropenia (44%) were the most common AEs, and neutropenia was the most common grade 3–4 AE (31%) [45]. A phase II trial from the MD Anderson Cancer Center trials group is currently assessing the efficacy and safety of zanu in association with rituximab in previously untreated patients with CLL/SLL (NCT04458610). Another phase II study is also assessing the safety and efficacy of zanu with tafasitamab (a humanized monoclonal anti-CD19 antibody, recently approved by the FDA) in combination with lenalidomide for R/R diffuse large B-cell lymphoma (DLBCL) not eligible for stem cell transplantation (TaZa CLL Study; NCT05718869).

ZV was studied in Cohort 3 of the SEQUOIA trial to treat patients with TN CLL/SLL who had the del(17p) mutation [32], and early results suggest that this combination was effective and well tolerated. Another open-label, non-randomized phase II trial is assessing ZV in patients with CLL/SLL who have relapsed after at least one prior therapy. In this trial, patients have been stratified into three groups: Cohort A, patients who have never received a BTK or BCL-2 inhibitor; Cohort B, patients who have received prior treatment with a BTK or BCL-2 inhibitor and discontinued treatment for any reason other than disease progression; and Cohort C, which includes patients who have experienced disease progression while treated with a prior BTK inhibitor (NCT05168930)).

### 4.4. Three Drug Zanubrutinib Combinations

The three-drug combination of zanu, obinutuzumab, and venetoclax (BOVen) was investigated in 37 TN CLL patients [46]. This regimen, which included a 2-month lead-in with zanu and obinutuzumab prior to the commencement of venetoclax, was attractive in that the treatment duration was limited to between 8 and 24 cycles of therapy. The duration of BOVen administration was determined according to the timing of the attainment of uMRD in the peripheral blood and bone marrow. After a median of 25.8 months follow-up, 33 (89%) of 37 patients had uMRD and thus met the predetermined criteria for therapy discontinuation. Thrombocytopenia (59%) was the most common AE of any grade, followed by fatigue (54%) and neutropenia (51%), while easy bruising (51%) was the most common grade 3 or worse AE. A patient died of an intracranial hemorrhage on day 1 of cycle 1 after intravenous heparin had been administered for the treatment of concurrent pulmonary embolism.

In comparison, patients in a phase II, single-arm, AVO (acalabrutinib/venetoclax/obinutuzumab) study also had comparable uMRD rates to those observed with BOVen (uMRD in peripheral blood and in bone marrow, 92% and 86%, respectively) [47]. However, the novel aspect of the BOVen study described above was the incorporated analysis of early MRD kinetics as a potential predictor for patients who may be more likely to respond to a shorter period of therapy [46]. The reduction in MRD by 400-fold beneath the baseline (MRD400) within the first 4 months of treatment was identified as the optimal threshold to define uMRD. Among 21 patients who achieved MRD400, 95% (20/21) required fewer than 12 cycles of therapy (median eight cycles) to achieve uMRD. On the other hand, among the 14 patients who failed to attain MRD400 after four months of BOVen, 50% (7/14) required more than 12 months of therapy (median 13 cycles). It is of interest to note that the recurrence of detectable MRD after one year (with 10^−5^ sensitivity) was only 5% in those who attained MRD400 and had discontinued therapy. In contrast, the 1-year MRD recurrence rate was 75% in patients who did not achieve MRD400 but had discontinued therapy after achieving the MRD endpoint [46]. These findings suggest that MRD400 may be a useful surrogate to recognize a subset of “earlier” responders whose clinical outcome is highly favorable once uMRD is achieved.

### 4.5. Mechanisms of Zanubrutinib Resistance in CLL

The dominant molecular mechanism associated with clinical resistance and loss of response to ibrutinib is the development of *BTK* Cys481 codon mutations [48]. Whether a similar mechanism mediates clinical resistance to the next-generation, more selective BTKi zanu is as yet unknown.

Of the six patients with zanu resistance who were available for longitudinal, targeted next-generation sequencing (NGS) and the dynamic assessment of clonal evolution, *TP53, EGR2, NOTCH1*, and *SF3B1* were the predominant genes associated with the development of resistance. Two patients developed emergent clones associated with *TP53* mutations at the point of progression, while another two patients showed persistence of *TP53* mutated clones. *SF3B1, EGR2,* and *BIRC3* mutated CLL subclones were stable during the development of clinical zanu resistance, while *BTK* Cys481 mutation (a secondary drug resistance mechanism), evolved during zanu treatment with clonal expansion due to positive clone selection [49].

Recently, Blombery et al. [50] analyzed the overall genetic landscape of *BTK* resistance mutations in patients who experienced disease progression during zanu treatment. The authors noted that *BTK* Leu528Trp mutations (also observed in patients with disease progression during treatment with pirtobrutinib), occurred more frequently in patients treated with zanu compared to patients treated with ibrutinib (54%; vs. 4%; *p* = 0.001). The mutational landscape present in the context of BTK inhibitor therapy at loci other than *BTK* Cys481 remains a poorly studied field that is in need of further investigation [50].

### 4.6. Zanubrutinib in Patients at Risk of Cardiovascular Complications

In CLL, the current therapeutic approach is influenced by a number of considerations such as the somatic genetic risk profile, comorbidities, concomitant medications, patient adherence, some logistical issues, and, most importantly, patient preference [51]. Patients with CLL are diagnosed at a median age of 72 years and often have an associated high prevalence of comorbidities, which may determine the therapeutic approach and choice of therapy [52]. As recently reported in a large retrospective cohort of patients mainly treated with BTKis, the three coexisting conditions most relevant for survival outcomes are the presence of any cardiovascular disorders, moderate/severe endocrine conditions, and upper gastrointestinal comorbidities [53].

Historically, patients with significant cardiovascular issues have generally been considered poor candidates for ibrutinib therapy; however, with the availability of second-generation BTKi therapies, more of these patients may be able to take advantage of treatment with BTK inhibition [54]. Overall, a multidisciplinary approach is most important to assess the eligibility of CLL patients for BTKi therapy. Particularly relevant aspects to consider include a history of valvular heart disease or other disorders that may increase the risk of AF; and a history of ventricular arrhythmias, clinical heart failure or left ventricular dysfunction, and reduced cardiac ejection fraction [55]. As a recent international panel of experts has suggested, the quantification of the cardiovascular (CV) risk posed by BTKis is important, as well as the interaction of BTKi therapy [56]. BTKi treatment should generally be avoided in patients with clinically significant heart failure (left ventricular ejection fraction of less than 30%). Furthermore, both ibrutinib and acalabrutinib should not be used in patients with a history of ventricular arrhythmias or in those with a family history of sudden cardiac death. It is of interest to note that among patients without prior ibrutinib exposure and in the absence of coronary disease or heart failure, the weighted average incidence of ventricular arrhythmias with acalabrutinib is 394 per 100,000 person years [57]. In comparison, there are 596 ibrutinib-related ventricular arrhythmias per 100,000 person years and 48.1 such events per 100,000 person years among similarly aged non-BTKi-treated subjects [58]. As yet, the comparable risks for zanu are unknown.

For patients with CLL in need of treatment at intermediate- or low-cardiovascular risk, second-generation BTKis such as acalabrutinib and zanu, are preferred. In the ASPEN study, patients with WM, treated with ibrutinib, had significantly more AF events of any grade (15%) than those treated with zanu (2%; *p* = 0.0004), while AF events of grade ≥3 were also significantly more common in the ibrutinib group (4% vs. 0%; *p* = 0.02). In addition, hypertension was more common in the ibrutinib-treated group than in the zanu group, but this difference was not statistically significant [18]. It is noteworthy that in the ALPINE trial, the rates of hypertension were similar in the zanu (21.9%) and ibrutinib (19.8%) arms [20]. As yet, it is unclear if these results are due to differences in the sample population or related to other factors; however, this is an intriguing observation, and patients with CLL treated with zanu should be monitored for hypertension.

**Figure 2:**
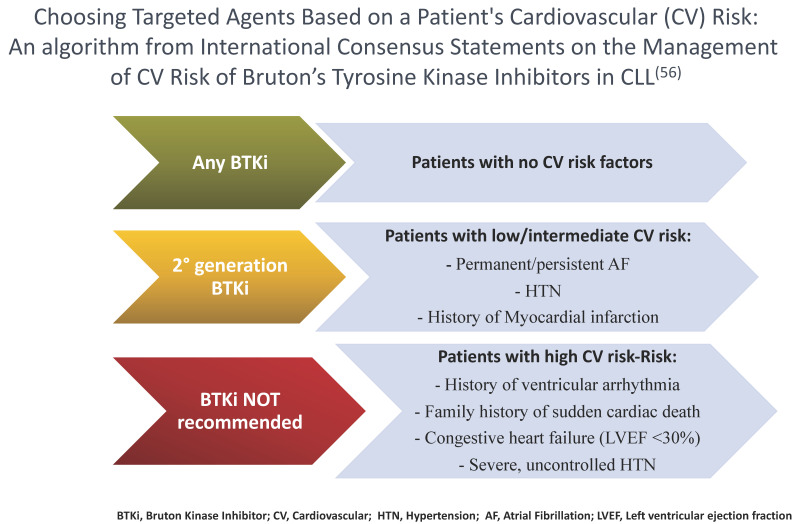
Representation of factors that may determine the choice of BTK inhibitor therapy based upon cardiovascular risk (CV) (^1^ [56]).

In conclusion, depending on the severity of cardiac comorbidities, treatment with a second-generation BTKi such as zanu can be challenging (Figure 2). Nonetheless, clinicians should be aware that effective cardiac screening and monitoring of cardiovascular complications must also be undertaken for patients at risk for cardiovascular toxicities who are treated with second-generation BTKis [56].

## 5. Conclusions

Updated NCCN (v2.2023) guidelines and the most recent German CLL algorithm both recommend second-generation BTKis, such as zanu and acalabrutinib, for the treatment of TN and R/R CLL regardless of patient fitness due to their increased selectivity and favorable drug toxicity profiles [23,24]. A recent comprehensive review and meta-analysis compared treatment-emergent AEs reported in clinical trials of ibrutinib, acalabrutinib, and zanu. A total of 61 trials involving 6959 patients treated with ibrutinib, acalabrutinib, and zanu were included. Overall, results from this meta-analysis show an improved AE profile for acalabrutinib and zanu compared to ibrutinib. Notably, zanu and acalabrutinib have a similar incidence of all grade (RR, 1.12) and grade ≥3 (RR, 0.90) AEs [59]. Therefore, the choice between these two different second-generation BTKis is driven predominantly by specific toxicity profiles and safety in older patients with comorbid conditions and cardiovascular risk factors [60], keeping in mind that some comorbidities may amplify toxicities related to a given BTKi. For example, in patients with a significant history of headaches, therapy with acalabrutinib may be less preferable [61]. According to recent data, tablet acalabrutinib formulations were similar to capsules, with the added benefit that tablets could be given together with PPIs without affecting pharmacokinetics and pharmacodynamics [62].

As yet we do not have robust evidence to suggest that second-generation BTKis can modify the natural course of CLL in specific genetic subgroups, but recent preliminary observations are of interest [63]. A pooled analysis of two clinical studies (ELEVATE-TN and CL-003), designed to compare PFS and OS for acalabrutinib combined with obinutuzumab versus acalabrutinib monotherapy in patients with TN CLL, clearly showed the benefit of adding obinutuzumab to acalabrutinib monotherapy across genomic subgroups, particularly in those with unmutated *IGHV* or without del(17p)/*TP53* mutations or complex karyotype abnormalities [64]. In the ALPINE trial, involving CLL patients with 17p deletion, *TP53* mutation, or both, zanu showed improved survival outcomes compared to ibrutinib. This observation was not evident in the ELEVATE-RR trial, which compared acalabrutinib and ibrutinib [11].

Major differences in populations enrolled in the ELEVATE-RR and ALPINE trials do not enable them to be compared. This does not apply to the ASCEND trial that assessed acalabrutinib in a patient population of R/R CLL patients similar to that included in the ALPINE trial [65]. The results of an unanchored matching-adjusted indirect comparison (MAIC) comparing the efficacy and safety of acalabrutinib vs. zanu, using individual patient data (IPD) from ASCEND and published aggregate data from ALPINE, have recently been presented. In this indirect comparison, acalabrutinib and zanu were shown to have a similar efficacy in patients with RR CLL (PFS at 24 months 76% and 78%, respectively) [66].

Understanding the “dead-kinase” mutations at codon L528 of BTK, which have been linked to disease progression on zanu and pirtobrutinib, may be relevant in this respect [50]. These have been reported less frequently with ibrutinib or acalabrutinib. For patients with double-class refractory diseases who have few alternatives for successful therapy, these mutations may have implications. Following Zanu, dead-kinase L528 mutations may result in pirto cross-resistance [50]. However, the true prevalence of L528 BTK mutations developing after Zanu treatment is still unknown and should not be a deciding factor in choosing a first BTK inhibitor until additional information is available.

Finally, the CAPTIVATE and GLOW trial results led to the EMA approval of the ibrutinib–venetoclax combination [67,68], while emerging data indicate that second-generation more selective BTKis, such as zanu, may well provide a valid alternative to ibrutinib in doublet regimens combining a BTKi with BCL2i. In this respect, preliminary safety data suggest that the combination regimen of ZV is generally well tolerated in this high-risk population, with no new safety signals identified [32]. The results of the long-term follow-up of arm D of the SEQUOIA trial are eagerly awaited.

In light of all the above, we feel that zanu represents an important landmark development in the treatment of CLL and is an exciting addition to the clinician’s therapeutic armamentarium and an attractive option for patients with CLL needing therapy (Table 1).

## Figures and Tables

**Table 1:** Efficacy and safety results of zanubrutinib in clinical trials.

Reference	Schedule	N of Pts (Tx Status)	17p(del)/TP53 Mut (%)	ORR (%)	Survival Outcome	G ≥ 3 AEs (%)	Toxicity-related Discontinuations (%)	Any Grade/(G ≥ 3) AF (%,)	Any Grade Bleeding/ (G ≥ 3) (%)	Any Grade Hypertension/ (G ≥ 3) (%)
Cull et al. [27]	Zanu 160 mg bid or 320 mg/d or 160 mg/d	22 (TN) 101 (R/R)	5.6 (TN) 6.0 (R/R)	100 (TN) 95 (R/R)	3-YEAR PFS: 90 (TN) 81 (R/R)	73.2	9.8	4.9 (3.3)	38.2 (3.3)	19.5 (8.9)
Tam et al. [19] (SEQUOIA Group A)	Zanu 160 mg bid	241 (TN)	1	94.6	2-YEAR PFS: 85.5%	53	8	3	41 (3.7)	6 (6)
Tam et al. [31] (SEQUOIA Group C)	Zanu 160 mg bid	111 (TN)	99	90	2-YEAR PFS: 88.9%	55	5		48 (5)	5 (5)
Brown et al. [20] 2022	Zanu 160 mg bid Ibrutinib: 420 mg/d	327 (R/R) 325 (R/R)	13.8 15.4		2-YEAR PFS: 78.4 65.9	67.3 70.4	16.2 22.2	6.2 (2.5) 13.3 (4)	42.3 (3.4) 41.4 (3.7)	23.5 (15.1) 22.8 (13.6)

Tx: treatment; G: grade; ORR, overall response rate; AEs: adverse events; AF; atrial fibrillation; bid: twice daily; d: daily; C: cycle; TN: treatment naïve; R/R: relapsed/refractory.
